# Supplementary material for: Telomeres as integrative markers of exposure to stress and adversity: a systematic review and meta-analysis
Source: R Soc Open Sci. 2018 Aug 29;5(8):180744. doi: 10.1098/rsos.180744 (PMC6124068; doi:10.1098/rsos.180744)
Supplement: PRISMA diagram [file rsos180744supp1.pdf]

PRISMA Flow Diagram

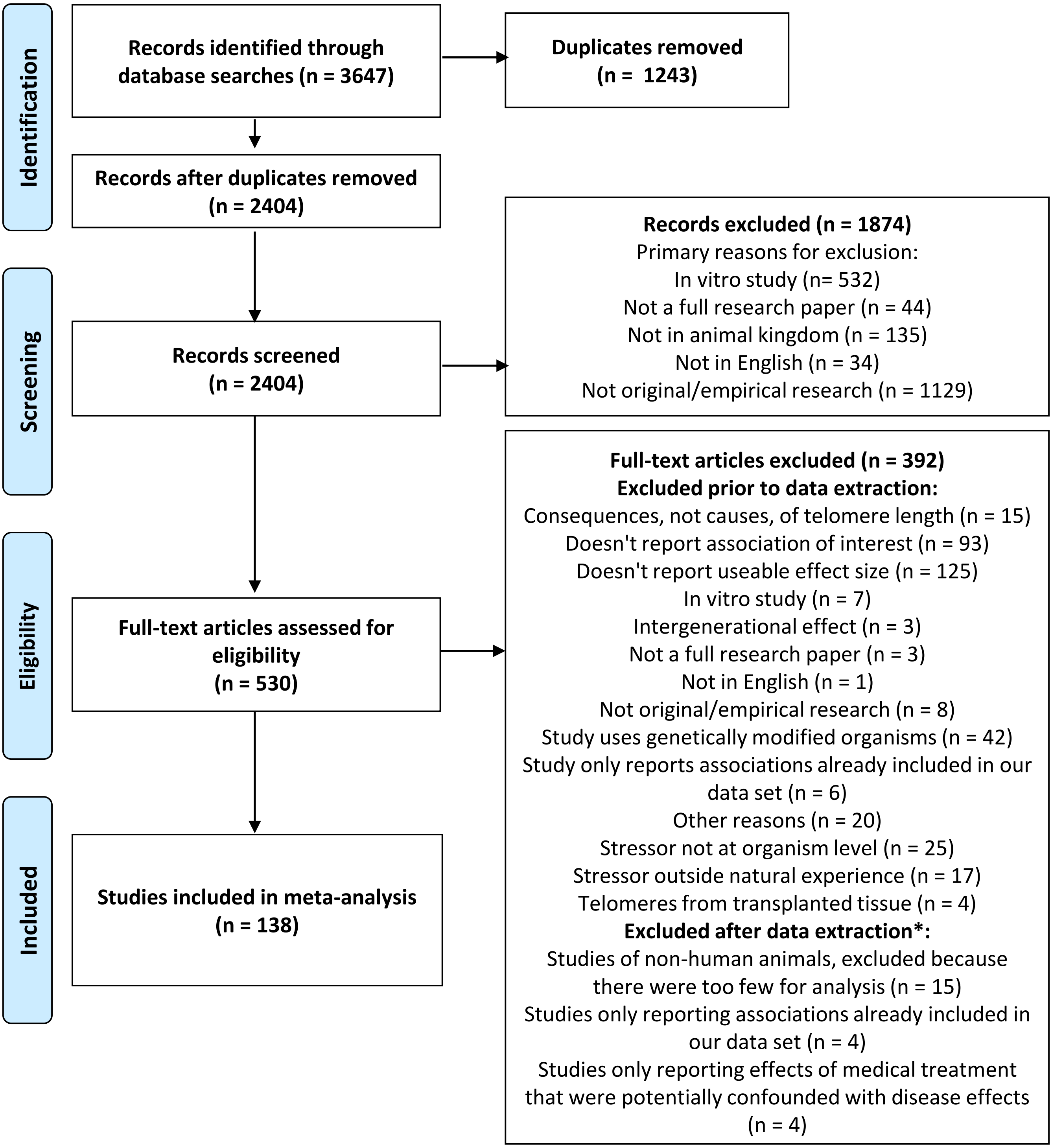

Note that sample sizes shown in this diagram refer to numbers of papers, rather than numbers of associations. In some cases, studies were excluded for multiple reasons. For simplicity, only the primary reason for exclusion is shown. *For these cases associations were extracted and are available in the unprocessed data file in our data archive, but were excluded from final analyses and processed data file for the reasons shown.
