# Supplementary material for: Telomeres as integrative markers of exposure to stress and adversity: a systematic review and meta-analysis
Source: R Soc Open Sci. 2018 Aug 29;5(8):180744. doi: 10.1098/rsos.180744 (PMC6124068; doi:10.1098/rsos.180744)
Supplement: Supplementary analyses [file rsos180744supp2.pdf]

Supplementary analyses for Pepper, Bateson and Nettle 'Telomeres as integrative markers of exposure to stress and adversity: A systematic review and meta-analysis'

1. Background to the supplementary analyses

Certain statistical analyses used to detect outliers and publication bias cannot be straightforwardly applied to our dataset due to its multilevel structure (multiple studies each contribute multiple non-independent associations). The statistical methods required (using 'metaplus' and 'weightr' packages in R) are not currently implemented for this kind of data structure. One possibility would be to ignore the multilevel structure for these particular analyses. However, in our view this would be misleading, since most of the variation in our dataset resides at the between-study level; the multiple associations from the same study are generally highly correlated with one another ($\rho = 0.87$ for the simplest main analysis). Thus, to treat each association as statistically independent would be to pseudo-replicate the information from certain studies many times. In a simple meta-analytic model with no moderators, the central estimate of association is $r = -0.15$ (95% CI -0.18 to -0.11) when the multilevel structure is properly accounted for, but $r = -0.09$ (95% CI -0.10 – -0.07) if the multilevel structure is ignored and a simple random effects model is fitted. The reason for this attenuation of association strength is that studies that contribute more associations to the dataset also contribute associations that are closer to zero on average (correlation between number of associations reported and the absolute value of the mean correlation coefficient reported, $r_{136} = -0.27$, $p < 0.01$). Thus, treating each of their data points as independent increases the influence of weak or null associations on the overall estimate.

An alternative approach that we use here is to create a 'flat' version of the dataset, in which one of the reported associations is chosen at random for each of the 138 studies. This produces a dataset with no multilevel structure, suitable for use with R packages 'metaplus' and 'weightr'. Given that the multiple associations from the same study tend to be similar to one another, a simple random effects model of the 'flat' dataset leads to inferences that are broadly similar to the multilevel model of the full dataset. For example, figure S1 shows the central estimate of association between exposures and telomeres from the main multilevel model, and from ten runs of the 'flat' sampling procedure. The similarity suggests that, where it is not possible to use the full dataset, analyses of a 'flat' sample are fairly informative.

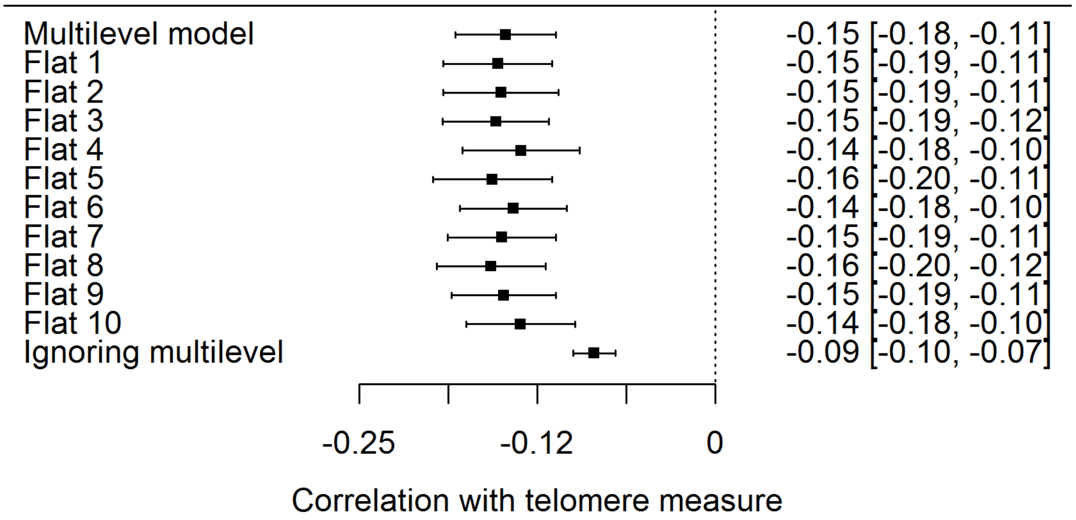

Figure S1. Central estimates of association between exposures and telomere measures for the main dataset analysed with a multilevel model, for ten runs of the 'flat' sampling procedure (see text), and for a model of the full dataset that ignores the multilevel structure and treats every association as independent.

2. Analysis of outliers

The standard random-effects model in meta-analysis assumes that the study-level random effects are Normally distributed. In practice, however, there may be a subset of outlier studies that depart from the central association more extremely than this. The R package 'metaplus' allows for the detection of the existence of such outliers. It fits a model in which the random effects are assumed to be drawn from a mixture of two distributions, one distribution for typical studies, and another, with a larger dispersion, for outlier studies (Beath, 2016). The fit of this mixture model can then be compared to the standard random-effects model: if there are studies that are marked outliers, the Bayesian Information Criterion (BIC) will be lower for the mixture model than the standard model. The studies most likely to belong to the outlier distribution can also be identified. We fit the mixture model, and a standard random-effects model, in ten 'flat' samples of the dataset (see section 1). Note that this procedure has limitations: a study *all* of whose associations are extreme will consistently be identified as an outlier, but a study reporting many associations only one of which is extreme will not. This should be borne in mind in what follows.

The fit for the mixture model was better than the standard model in all ten samples (mean BIC, mixture model: -24.33; standard model: -7.34). Within the mixture model, the estimated dispersion of true effects was much larger for the studies assigned to the outlier set than for the rest of the studies (mean τ, outliers: 0.35, typical: 0.07). The mean central estimate of association between exposures and telomeres for the mixture model was r = -0.09, somewhat weaker than the estimate from the standard model of r = -0.15. Thus, this analysis suggests that there is a set of outlier studies

with atypically strong associations, whose aggregate effect is to make the negative correlation between exposures and telomeres appear stronger than the remaining studies would.

We examined which studies were assigned an average posterior probability of 0.9 or more of belonging to the outlier set (table S1). The 12 studies identified as outliers tended to be small-*n* (median *n* 91), and mostly reported strong negative associations between exposures and TL (10/12; the remaining two reported stronger-than-expected associations in the opposite direction). Four studies of diabetes appeared on this list, suggesting that diabetes may have genuinely stronger associations with telomere length than other categories of exposure do. Two substantial studies of sleep apnea also appeared on the list, though their findings were in opposite directions to one another, one of children suggesting longer telomere length in sleep apnea, and one in adults suggesting shorter telomere length in sleep apnea. Many of the remaining studies in table S1 had such small samples that the extreme results might simply reflect sampling variability.

Table S1. Studies assigned to the outlier set in the mixture-model analysis with an average posterior probability of 0.9 or greater. Note that only the first author is shown in the 'Study' column; for full study titles, please see processed data file in the online data archive.

Study	Broad category	N	Comments
Adaikalakoteswari (2005)	Disease	80	Small study of type-2 diabetes, strong negative association of TL with disease status (patients shorter TL)
Castaldo (2013)	Disease	37	Small study on Friedrich's ataxia, strong negative correlation of TL with duration of disease (longer duration shorter TL)
Defelice (2012)	Environmental hazard	50	Small study, very strong negative association of TL with proximity to toxic waste sites (closer to pollution shorter TL)
Gardner (2005)	Disease	70	Small longitudinal study of type-2 diabetes, strong association between change in insulin resistance and change in TL (greater rise in insulin resistance, greater TL attrition)
Hudson (2011)	Disease	208	Study of Parkinson's disease; substantial association between white blood cell TL and disease status in opposite direction to expectation (patients longer TL)
Kim (2010)	Disease	213	Study of obstructive sleep apnea in children; substantial association between TL and sleep apnea severity in opposite direction to expectation (worse severity longer TL)
Krishna (2015)	Psychosocial	33	Small study of yoga practitioners; very large TL difference between practitioners (longer TL) and controls (shorter TL). NB. These results would be less extraordinary, though still unusually strong, if what are described as standard deviations in table 1 of the paper were actually standard errors.
Ma (2013)	Disease	102	Study of diabetes; strong negative associations between disease status and TL (patients shorter TL)
Marchetto (2016)	Psychosocial	24	Small study of prenatal stress and TL in newborn babies; strong negative association between maternal stress and TL in newborns (higher maternal stress shorter newborn TL)
Monickaraj (2012)	Disease	290	Study of type-2 diabetes, strong negative association between disease status and TL (patients shorter TL)
Savolainen (2014b)	Disease	1948	Large study of sleep apnea; substantial negative correlation between disease status and TL (patients shorter TL)
Snetselaar (2015)	Disease	532	Study of interstitial lung disease, strong negative associations between disease status and TL (patients shorter TL)

3. Further analyses of publication bias

The model of Vevea and Hedges (1995) allows for assessment of, and correction for, different types of publication bias. The model simultaneously estimates the relative probability of observation of associations that lie within specified *p*-value bands (such as $p < 0.05$ versus $p >= 0.05$), as well as the overall meta-analytic association adjusted for the differential probability of observation of associations in the given bands. The model is implemented in R package 'weightr'. We produced 10 'flat' samples of our dataset (see section 1), and fitted a standard random-effects model, plus the Vevea and Hedges model, specifying the following *p*-value regions: significant association ($p < 0.05$) in the opposite direction to the hypothesis that stress and adversity shorten telomeres; non-significant trend against the hypothesis; significant trend in the direction of the hypothesis; and significant association ($p < 0.05$) in the direction of the hypothesis. The mean unadjusted association was $r = -0.15$, as expected, but the mean after adjustment for publication bias was $r = -0.03$. Thus, this analysis supports the suggestion we make in the main paper that there may be publication bias, and that it may lead to an inflated estimate of the strength of the negative association between exposures and telomere measures.

The mean relative probabilities of observation were 8.37 for a non-significant trend against the hypothesis; 21.25 for a non-significant trend in the direction of the hypothesis; and 12.94 for significant association in support of the hypothesis. These numbers are relative to a significant association contrary to the hypothesis. That is, 12.94 indicates that a significant association in the direction of the hypothesis is 12.94 more times more likely to be published, and hence appear in the dataset, than a significant association contrary to the hypothesis. The fact that all the numbers are greater than 1 suggests that every other type of result is more likely to be published than a significant finding that stress and adversity are associated with longer telomeres. In small samples, such associations should be found from time to time, because the true associations are weak, and precision of measurement is low. It is possible that researchers dismiss them as implausible or anomalous when they are found, and this explains their rarity in the dataset. This analysis suggests that the publication bias issue in this literature is not so much differential suppression of non-significant results (after all, the category with the highest relative probability of observation is actually non-significant trends in the direction of the hypothesis), but differential suppression of results that go significantly contrary to expectation.

Figure S2 visualizes this pattern. It repeats figure 1b of the main paper, but with points coloured by whether the original authors reported the associations as significant or not. As can be seen, there are plenty of non-significant associations, especially where their direction is as expected. However, there is a marked paucity of significant associations in the positive direction, even though the very broad width of the triangle where *n* is small suggests that these ought to exist.

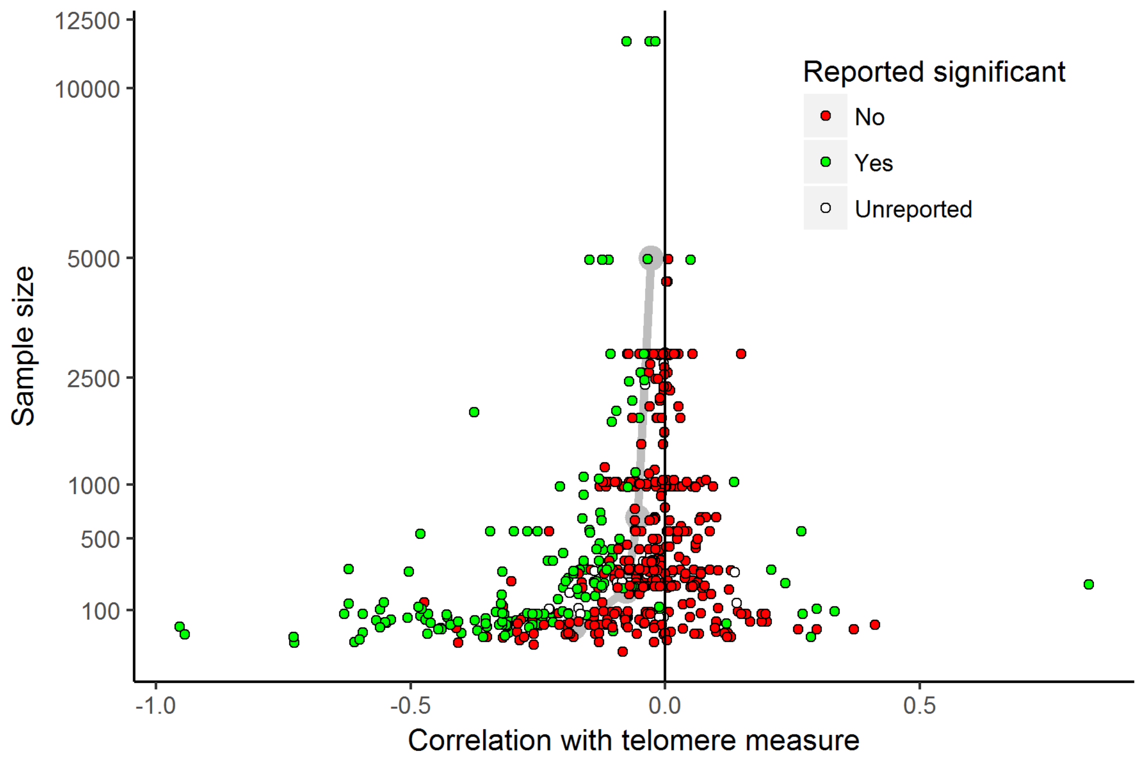

Figure S2. Funnel plot of sample size against observed correlation between telomere measure and exposure variable, with associations coloured to indicate whether the original authors reported them as significant or not.

References

Beath, K. J. (2016). metaplus: An R package for the analysis of robust meta-analysis and meta-regression. *The R Journal*, *8*, 5–16.

Vevea, J. L., & Hedges, L. V. (1995). A general linear model for estimating effect size in the presence of publication bias. *Psychometrika*, *60*, 419–435. http://doi.org/10.1007/BF02294384
